# Quantum Efficiency Enhancement of a GaN-Based Green Light-Emitting Diode by a Graded Indium Composition p-Type InGaN Layer

**DOI:** 10.3390/nano8070512

**Published:** 2018-07-09

**Authors:** Quanbin Zhou, Hong Wang, Mingsheng Xu, Xi-Chun Zhang

**Affiliations:** 1 Engineering Research Center for Optoelectronics of Guangdong Province, School of Electronics and Information Engineering, South China University of Technology, Guangzhou 510640, China; zhouquanbin86@163.com (Q.Z.); mshxu@163.com (M.X.); 2 School of Physics and Optoelectronics, South China University of Technology, Guangzhou 510640, China

**Keywords:** p-type InGaN, graded indium composition, hole injection, quantum efficiency, green LED

## Abstract

We propose a graded indium composition p-type InGaN (p-InGaN) conduction layer to replace the p-type AlGaN electron blocking layer and a p-GaN layer in order to enhance the light output power of a GaN-based green light-emitting diode (LED). The indium composition of the p-InGaN layer decreased from 10.4% to 0% along the growth direction. The light intensity of the LED with a graded indium composition p-InGaN layer is 13.7% higher than that of conventional LEDs according to the experimental result. The calculated data further confirmed that the graded indium composition p-InGaN layer can effectively improve the light power of green LEDs. According to the simulation, the increase in light output power of green LEDs with a graded indium composition p-InGaN layer was mainly attributed to the enhancement of hole injection and the improvement of the radiative recombination rate.

## 1. Introduction

GaN-based light-emitting diodes (LEDs) have attracted considerable attention and have been seen as a promising replacement for conventional light sources in the last few decades [1,2]. The efficiency of blue LEDs is very high, and blue LEDs have been commercially used in many fields, such as lighting [3,4,5,6], display [7,8], light communication [9,10], back lighting [11,12], and so on. However, the internal quantum efficiency (IQE) of GaN-based green LEDs is still lower than that of blue LEDs, which is called the “Green Gap” [13]. It obstructs the green LED to be applied in Red-Green-Blue (RGB) lighting, full-color displays, and visible-light communication. A large polarization field [14,15,16] and poor crystal quality [17,18] are the main reasons for the low IQE of green LEDs with a high indium composition. In fact, the poor hole injection also plays an important role in the low quantum efficiency of GaN-based LEDs. Many researchers have proposed various methods to solve this problem based on band engineering of the electron blocking layer (EBL). Kim et al. employed an active-layer-friendly lattice-matched InAlN EBL to improve the quantum efficiency of green LEDs [19]. A graded superlattice AlGaN/GaN inserting layer was proposed by J. Kang et al. to enhance the efficiency of hole injection and performance of green LEDs [20]. An InAlGaN/GaN superlattice [21], an AlGaN/InGaN superlattice [22,23], and a composition-graded AlGaN EBL [24,25,26] were also employed to reduce the potential barrier of holes without damaging the electron confinement. A recently proposed method to improve the properties of p-type GaN is polarization doping [27]. It uses the internal polarization of the structures and material composition grading to induce free electrons or holes [28]. However, the growth temperature of AlGaN is always high in order to improve the crystal quality. The high indium content InGaN/GaN multiple quantum well (MQW) of green LEDs will be damaged during the high temperature process [29,30,31,32]. There are few reports about the p-type layer structure designed to improve the hole injection of GaN-based LEDs [33].

In this paper, we designed a new structure of p-type InGaN (p-InGaN) conduction layer with a graded indium composition to replace the conventional p-type AlGaN (p-AlGaN) EBL and p-type GaN (p-GaN) conduction layer of GaN-based green LEDs. The effect of the graded indium composition p-InGaN conduction layer on the light output power of green LEDs is studied by experiments and simulations.

## 2. Experimental Details

**Figure 1:**
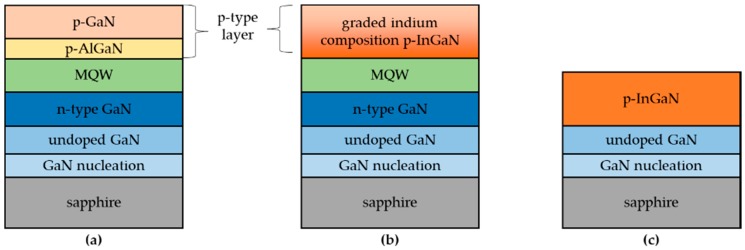
Schematic diagrams for the LED and p-InGaN samples: (**a**) sample A; (**b**) sample B; and (**c**) sample C.

The LED samples were grown on (0001)-oriented sapphire substrates by an AIXTRON close-coupled showerhead metal-organic chemical vapor deposition (MOCVD) reactor (MOCVD, AIXTRON Inc., Herzogenrath, Germany). The trimethylgallium (TMGa), trimethylaluminum (TMAl), trimethylindium (TMIn), and ammonia (NH_3_) were used as sources of gallium, aluminum, indium, and nitrogen, respectively. Silane (SiH_4_) and bicyclopentadienyl magnesium (Cp_2_Mg) were used as n-type and p-type doping sources, respectively. The epitaxial structure of conventional LEDs consisted of a 30 nm thick GaN nucleation layer grown at 530 °C, a 3 µm thick undoped GaN (u-GaN) buffer layer grown at 1100 °C, a 4 µm thick Si-doped n-type GaN (n-GaN) layer with 8 × 10^18^ cm^–3^ doping concentration grown at 1080 °C, five pairs of 3 nm and 10 nm thick In_0.22_Ga_0.78_N/GaN MQWs active layers, a 20 nm thick p-type Al_0.15_Ga_0.85_N (p-AlGaN) electron blocking layer grown at 1040 °C, and a 180 nm thick p-GaN layer grown at 940 °C. The doping concentration of the p-AlGaN and p-GaN layers was 5 × 10^18^ cm^–3^. The conventional LEDs were denoted as sample A. Figure 1a is the profile of sample A. We also prepared green LEDs with a graded indium composition p-InGaN conduction layer, which are denoted as sample B. The growth conditions of sample B were similar to that of sample A except for the p-type layers. As shown in Figure 1b, the p-AlGaN and p-GaN layers were replaced by a p-InGaN single layer with a thickness of 200 nm. The growth temperature of p-InGaN layer was 860 °C, and the flow of TMIn changed from 175 sccm to 0 sccm along the growth direction in order to obtain a p-InGaN layer with a graded indium composition. Furthermore, we grew another sample with only a p-InGaN film on u-GaN in order to determine the indium composition of the p-InGaN layer. The TMIn flow of the p-InGaN film remained at 175 sccm, and the growth temperature was 860 °C. Figure 1c shows the schematic diagram of the p-InGaN film, which is denoted as sample C. The thickness of the p-InGaN layer in sample C was 80 nm.

After the growth of the LED structure of sample A and sample B, the epitaxial wafers were treated together to LED chips. The epitaxial wafers were first cleaned in acetone, isopropanol, deionized water, and partly etched to n-GaN by an inductively coupled plasma (ICP) system. Next, these samples were cleaned by a sulfuric acid peroxide mixture at 60 °C, ammonia water at 35 °C, deionized water at room temperature, and dried by nitrogen gas. Sequentially, the 80 nm indium tin oxide (ITO) transparent conductive electrodes (TCEs) were evaporated by electron beam evaporation and then annealed at 600 °C for 3 min in a mixture of ambient N_2_/O_2_ (200:35). The ITO TCEs were selectively removed by wet chemical etching. Then, the SiO_2_ passivation layer was deposited by a plasma enhanced chemical vapor deposition (PECVD) system. Finally, Cr (50 nm)/Al (200 nm)/Ti (100 nm)/Au (100 nm) electrode layers were deposited by electron beam evaporation. The size of the LED chip was 1.14 mm × 1.14 mm.

## 3. Results and Discussion

**Figure 2:**
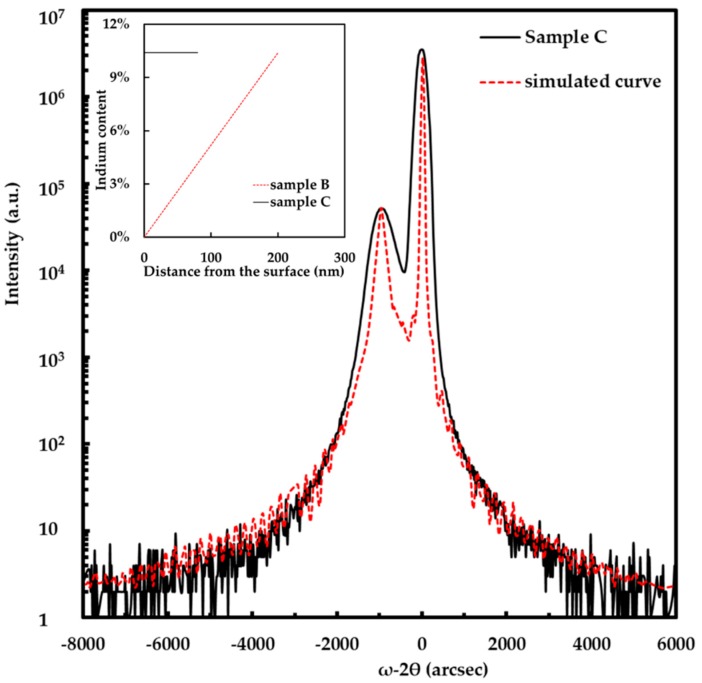
High-resolution X-ray diffraction ω-2θ scan of sample C. The dashed line in red is the simulated curve. The inset shows the indium content profiles of samples B and C.

We characterized the crystallography of the p-InGaN film in sample C by a Rigaku high-resolution X-ray diffraction (XRD, Rigaku Inc., Tokyo, Japan) with Cu K_α_ irradiation at 40 kV and 100 mA. Figure 2 is the HRXRD ω-2θ scan of p-InGaN film in sample C. The main peak and the secondary peak are GaN and InGaN, respectively. Chen et al. and Zhou et al. calculated the indium composition of the In*_x_*Ga_1−*x*_N film using Vegard’s law and the XRD data [34,35,36]. We evaluated the indium content of the p-InGaN film by the separation between GaN and InGaN peaks in the XRD spectra. The indium content of p-InGaN in sample C was 10.4%. No evidence of phase separation could be found in the XRD spectrum. When the indium content is not too high, it almost linearly increases as the TMIn flow increasing [37]. Because the p-InGaN in sample B had similar growth conditions to sample C, except for the TMIn flow changing form 175 sccm to 0 sccm, the indium content in p-InGaN of sample B was from 10.4% to 0% along the growth direction. The expected indium content profiles of samples B and C are shown in the inset of Figure 2.

**Figure 3:**
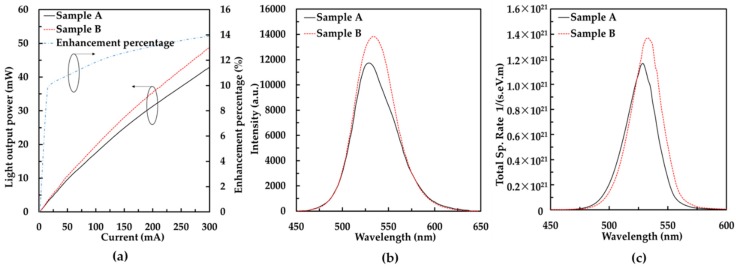
Light output properties of samples A and B: (**a**) measured light output power and enhancement percentage as a function of the injection current; (**b**) measured EL spectra at 300 mA; and (**c**) calculated EL spectra at 300 mA.

The light output properties of the two LED samples are shown in Figure 3. Figure 3a shows the light output power as a function of injection current. Sample B had a great enhancement of light output power for the whole injection current range compared with the sample A. The light output power of sample B was 13.7% larger than that of sample A at a 300 mA injection current. The result revealed that the graded indium composition p-InGaN conduction layer was beneficial for enhancing the light output power of a GaN-based green LED. It is clear that the peak intensity of electroluminescence (EL) spectra at 300 mA of sample B was stronger than that of sample A, as shown in Figure 3b. Furthermore, the peak wavelengths of samples A and B were 529 nm and 534 nm respectively. 

**Figure 4:**
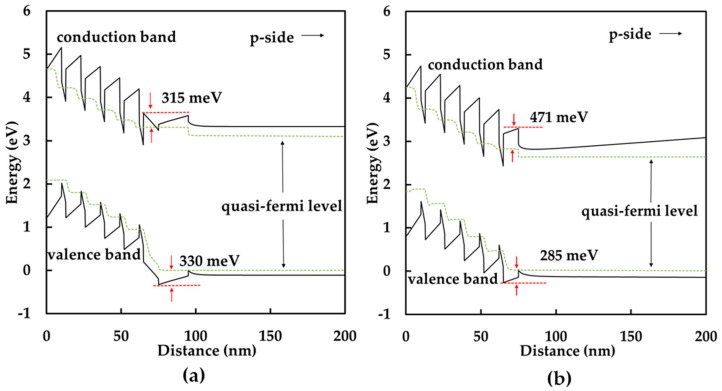
Calculated energy band diagrams at 300 mA of (**a**) sample A; and (**b**) sample B.

In order to better understand the influence of the graded indium composition p-InGaN conduction layer on the performance of GaN-based green LEDs, we performed a numerical simulation using APSYS software (version 2010, Crosslight Software Inc., Burnaby, BC, Canada). The software self-consistently solves the Poisson equation, continuity equation, and Schrödinger equation with boundary conditions [38,39]. Here, the band-offset ratio between the conduction band and the valence band for InGaN/GaN MQWs was 70% [40]. In addition, the values of the Shockley-Read-Hall (SRH) recombination lifetime and Auger coefficients assumed in this simulation were 50 ns and 1 × 10^−30^ cm^6^/s, respectively [41,42,43]. Figure 3c displays the calculated EL spectra at 300 mA, which shows a similar trend of enhancement in light intensity with measured results. The simulated energy band diagrams at 300 mA of sample A and sample B are shown in Figure 4. The solid lines are conduction bands and valence bands and the dashed lines are the quasi-fermi level. In sample A, the height of the barrier, which obstructs holes injecting into the MQW, is 330 meV. However, in sample B, the height of the barrier is 285 meV when the p-AlGaN EBL and p-GaN conduction layer are replaced by the graded indium composition p-InGaN conduction layer. The lower potential barrier is beneficial for holes injecting into the MQW.

**Figure 5:**
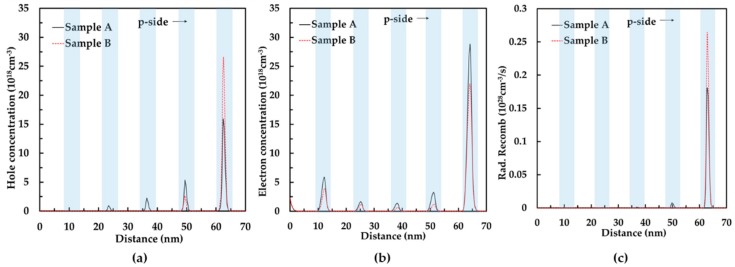
(**a**) Hole concentration distribution; (**b**) electron concentration distribution; and (**c**) radiation recombination rate distribution of samples A and B.

Figure 5 plots the carrier concentration distribution and radiation recombination rate distribution in the MQW of sample A and sample B from n-side to p-side. The holes of samples A and B both accumulate in the quantum well near the p-type layer. As shown in Figure 5a, the hole concentration in the quantum well near the p-type layer of sample B was much larger than that of sample A. The function of the EBL is to reduce the electron overflow leakage. Therefore, sample A had a little more electron concentration in the MQW, as shown in Figure 5b. The higher carrier concentration led to a larger radiation recombination rate of GaN-based LED. From Figure 5c, we could find that the radiation recombination rate of sample B was much bigger than that of sample A in the quantum well near the p-type layer. As a result, sample B had a higher total radiation recombination rate compared to sample A. The simulation data demonstrated that the graded indium composition p-InGaN conduction layer could enhance the hole injection and radiation recombination rate of GaN-based green LEDs.

## 4. Conclusions

In conclusion, the light output properties of GaN-based green LEDs with and without a graded indium composition p-InGaN layer were numerically and experimentally investigated. Both the experimental results and simulated data revealed that the graded indium composition p-InGaN conduction layer can promote the light output power of green LEDs. The light output power of green LEDs with a p-InGaN conduction layer was enhanced by 13.7% compared to the conventional LED, according to the experimental data. The simulation results demonstrated that the improvement in light output property was mainly due to the increase of hole injection and the enhancement of the radiative recombination rate.

## References

[1] Wang L., Liu Z., Zhang Z.H., Tian Y.D., Yi X., Wang J., Li J., Wang G. (2016). Interface and photoluminescence characteristics of graphene-(GaN/InGaN)_n_ multiple quantum wells hybrid structure. J. Appl. Phys..

[2] Wang Q., Ji Z., Zhou Y., Wang X., Liu B., Xu X., Gao X., Leng J. (2017). Diameter-dependent photoluminescence properties of strong phase-separated dual-wavelength InGaN/GaN nanopillar LEDs. Appl. Surf. Sci..

[3] Pimputkar S., Speck J.S., Denbaars S.P., Nakamura S. (2009). Prospects for LED lighting. Nat. Photonics.

[4] Sinnadurai R., Khan M.K.A.A., Azri M., Vikneswaran V. Development of White LED down Light for Indoor Lighting. Proceedings of the IEEE Conference on Sustainable Utilization and Development in Engineering and Technology (STUDENT).

[5] Moon S., Koo G.B., Moon G.W. (2013). A new control method of interleaved single-stage flyback AC–DC converter for outdoor LED lighting systems. IEEE Trans. Power Electron..

[6] Poulet L., Massa G.D., Morrow R.C., Bourget C.M., Wheeler R.M., Mitchell C.A. (2014). Significant reduction in energy for plant-growth lighting in space using targeted LED lighting and spectral manipulation. Life Sci. Space Res..

[7] Sanchot A., Consonni M., Calvez S.L., Robin I.C., Templier F. (2015). Color conversion using quantum dots on high-brightness GaN LED arrays for display application. MRS Proc..

[8] Lee K.J. (2012). Flexible GaN LED on a polyimide substrate for display applications. Proc. SPIE.

[9] Ferreira R., Xie E., Mckendry J., Rajbhandari S. (2016). High bandwidth GaN-based micro-LEDs for multi-Gb/s visible light communications. IEEE Photonics Technol. Lett..

[10] Du C., Huang X., Jiang C., Pu X., Zhao Z., Jing L., Hu W., Wang Z.L. (2016). Tuning carrier lifetime in InGaN/GaN LEDs via strain compensation for high-speed visible light communication. Sci. Rep..

[11] Anandan M. (2008). Progress of LED backlights for LCDS. J. Soc. Inf. Displ..

[12] Soon C.M. (2007). White Light Emitting Diode as Liquid Crystal Display Backlight.

[13] Zhou Q., Xu M., Wang H. (2016). Internal quantum efficiency improvement of InGaN/GaN multiple quantum well green light-emitting diodes. Opto-Electron. Rev..

[14] Young N.G., Farrell R.M., Iza M., Nakamura S., DenBaars S.P., Weisbuch C., Speck J.S. (2016). Germanium doping of GaN by metalorganic chemical vapor deposition for polarization screening applications. J. Cryst. Growth.

[15] Prajoon P., Nirmal D., Menokey M.A., Pravin J.C. (2016). Efficiency enhancement of InGaN MQW LED using compositionally step graded InGaN barrier on SiC substrate. J. Disp. Technol..

[16] Xu M., Yu W., Zhou Q., Zhang H., Wang H. (2016). Efficiency enhancement of GaN-based green light-emitting diode with PN-doped quantum barriers. Mater. Express.

[17] Ren P., Zhang N., Xue B., Liu Z., Wang J., Li J. (2016). A novel usage of hydrogen treatment to improve the indium incorporation and internal quantum efficiency of green InGaN/GaN multiple quantum wells simultaneously. J. Phys. D Appl. Phys..

[18] Qiao L., Ma Z.-G., Chen H., Wu H.-Y., Chen X.-F., Yang H.-J., Zhao B., He M., Zheng S.-W., Li S.-T. (2016). Effects of multiple interruptions with trimethylindium-treatment in the InGaN/GaN quantum well on green light emitting diodes. Chin. Phys. B.

[19] Kim H.J., Choi S., Kim S.-S., Ryou J.-H., Yoder P.D., Dupuis R.D., Fischer A.M., Sun K., Ponce F.A. (2010). Improvement of quantum efficiency by employing active-layer-friendly lattice-matched InAlN electron blocking layer in green light-emitting diodes. Appl. Phys. Lett..

[20] Kang J., Li H., Li Z., Liu Z., Ma P., Yi X., Wang G. (2013). Enhancing the performance of green GaN-based light-emitting diodes with graded superlattice AlGaN/GaN inserting layer. Appl. Phys. Lett..

[21] Lin D.-W., Tzou A.-J., Huang J.-K., Lin B.-C., Chang C.-Y., Kuo H.-C. Greatly improved efficiency droop for InGaN-based green light emitting diodes by quaternary content superlattice electron blocking layer. Proceedings of the International Conference on Numerical Simulation of Optoelectronic Devices (NUSOD).

[22] Yu C.-T., Lai W.-C., Yen C.-H., Chang S.-J. (2014). Effects of ingan layer thickness of AlGaN/InGaN superlattice electron blocking layer on the overall efficiency and efficiency droops of GaN-based light emitting diodes. Opt. Express.

[23] Chen F.-M., Liou B.-T., Chang Y.-A., Chang J.-Y., Kuo Y.-T., Kuo Y.-K. (2013). Numerical Analysis of Using Superlattice-AlGaN/InGaN as Electron Blocking Layer in Green InGaN Light-Emitting Diodes.

[24] Lei Y., Liu Z., He M., Yi X., Wang J., Li J., Zheng S., Li S. (2015). Enhancement of blue InGaN light-emitting diodes by using AlGaN increased composition-graded barriers. J. Semicond..

[25] Wang C.H., Ke C.C., Lee C.Y., Chang S.P. (2010). Hole injection and efficiency droop improvement in InGaN/GaN light-emitting diodes by band-engineered electron blocking layer. Appl. Phys. Lett..

[26] Kuo Y.K., Chang J.Y., Tsai M.C. (2010). Enhancement in hole-injection efficiency of blue InGaN light-emitting diodes from reduced polarization by some specific designs for the electron blocking layer. Opt. Lett..

[27] Kivisaari P., Oksanen J., Tulkki J. (2013). Polarization doping and the efficiency of III-nitride optoelectronic devices. Appl. Phys. Lett..

[28] Li S., Zhang T., Wu J., Yang Y., Wang Z., Wu Z., Chen Z., Jiang Y. (2013). Polarization induced hole doping in graded Al_*x*_Ga_1−*x*_N (*x* = 0.7~1) layer grown by molecular beam epitaxy. Appl. Phys. Lett..

[29] Oh M.S., Kwon M.K., Park I.K., Baek S.H., Park S.J., Lee S.H., Jung J.J. (2006). Improvement of green LED by growing p-GaN on In_0.25_GaN/GaN MQWs at low temperature. J. Cryst. Growth.

[30] Lee W., Limb J., Ryou J.H., Yoo D., Chung T., Dupuis R.D. (2006). Influence of growth temperature and growth rate of p-GaN layers on the characteristics of green light emitting diodes. J. Electron. Mater..

[31] Lee W., Limb J., Ryou J.H., Yoo D., Chung T., Dupuis R.D. (2006). Effect of thermal annealing induced by p-type layer growth on blue and green LED performance. J. Cryst. Growth.

[32] Ju J.W., Zhu J., Kim H.S., Lee C.R., Lee I.H. (2007). Effects of p-GaN growth temperature on a green InGaN/GaN multiple quantum well. J. Korean Phys. Soc..

[33] Lin Z., Wang H., Lin Y., Yang M., Li G., Xu B. (2016). A new structure of p-GaN/InGaN heterojunction to enhance hole injection for blue GaN-based LEDs. J. Phys. D Appl. Phys..

[34] Qin Z., Chen Z., Tong Y., Lu S., Zhang G. (2002). Estimation of InN phase inclusion in InGaN films grown by MOVPE. Appl. Phys. A.

[35] Chen Z.Z., Qin Z.X., Hu X.D., Yu T.J., Yang Z.J., Tong Y.Z., Ding X.M., Zhang G.Y. (2004). Study of photoluminescence and absorption in phase-separation InGaN films. Phys. B Condens. Matter.

[36] Zhou S.Q., Wu M.F., Hou L.N., Yao S.D., Ma H.J., Nie R., Tong Y.Z., Yang Z.J., Yu T.J., Zhang G.Y. (2004). An approach to determine the chemical composition in InGaN/GaN multiple quantum wells. J. Cryst. Growth.

[37] Guo Y., Liu X.L., Song H.P., Yang A.L., Xu X.Q., Zheng G.L., Wei H.Y., Yang S.Y., Zhu Q.S., Wang Z.G. (2010). A study of indium incorporation in In-rich InGaN grown by MOVPE. Appl. Surf. Sci..

[38] (2010). Apsys, Version 2010 Software for Electrical, Optical and Thermal Properties of Compound Semiconductor Devices.

[39] Zhao H., Arif R.A., Ee Y.K., Tansu N. (2008). Self-consistent analysis of strain-compensated InGaN-AlGaN quantum wells for lasers and light-emitting diodes. IEEE J. Quantum Electron..

[40] Li J., Guo Z., Li F., Lin H., Li C., Xiang S., Zhou T., Wan N., Liu Y. (2015). Performance enhancement of blue light-emitting diodes by using special designed n and p-type doped barriers. Superlattices Microstruct..

[41] Zhang M., Yun F., Li Y., Ding W., Wang H., Zhao Y., Zhang W., Zheng M., Tian Z., Su X. (2015). Luminescence properties of InGaN-based dual-wavelength light-emitting diodes with different quantum-well arrangements. Phys. Status Solidi.

[42] Cheng L., Wu S., Chen H., Xia C., Kong Q. (2016). Investigation of whether uniform carrier distribution in quantum wells can lead to higher performance in InGaN light-emitting diodes. Opt. Quantum Electron..

[43] Piprek J. (2010). Efficiency droop in nitride-based light-emitting diodes. Phys. Status Solidi.

